# The future of cardiac repair: a review on cell-free nanotherapies for regenerative myocardial infarction

**DOI:** 10.1007/s13346-024-01763-y

**Published:** 2025-01-20

**Authors:** Nermeen H. Kamal, Lamia A. Heikal, Ossama Y. Abdallah

**Affiliations:** 1 Department of Pharmaceutics, Division of Pharmaceutical Sciences. College of Pharmacy, Arab Academy for Science, Technology and Maritime Transport, Alexandria, Egypt; 2 Department of Pharmaceutics, Faculty of Pharmacy, Alexandria University, 1 Khartoum Square, Azarita, P.O. Box 21521, Alexandria, Egypt

**Keywords:** Cardiac regeneration, Passive targeting, Active targeting, Bioinspired, Biomaterial

## Abstract

Cardiovascular diseases as myocardial infarction (MI) represent a major cause for morbidity and mortality worldwide. Even though, patients who survive MI are susceptible to high risk of heart failure. This is mainly attributed to the major loss of cardiomyocytes and limited regenerative potential of myocardium. Despite the availability of various cardiovascular drugs, they fail to address the main cause of MI. The optimum therapeutic goal should therefore focus on enhancing cardiac regeneration through cellular and cell-free therapeutic approaches. This review focused on different mechanisms of cardiac regeneration that can be achieved via non-cellular therapeutic modalities. Passive and active targeting of the infarcted myocardium using various nanoparticles that can be loaded with growth factors, drugs or affordable natural products can reduce negative ventricular remodeling, infarct size and the apoptotic rate of cardiomyocytes. In addition, injectable biomaterials-based nanocomposite can be used as a scaffold to support infarcted heart and recruit cells. Innovative affordable and less invasive cell-free approaches can be implemented to enhance cardiac regeneration post MI.

**Graphical abstract:**

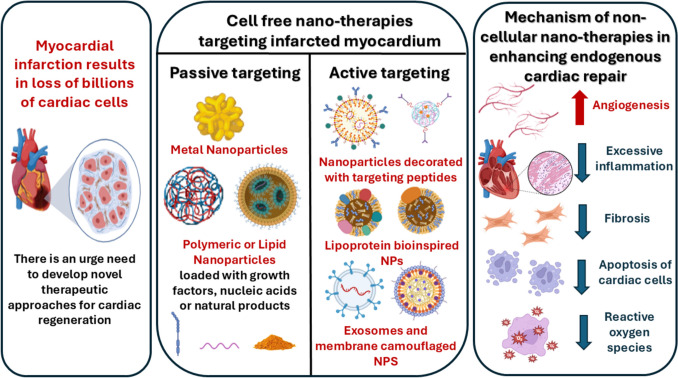

## Introduction

### Myocardial infarction: Prevalence, types, and pathophysiology

Myocardial infarction (MI) is the most severe type of cardiovascular diseases that is accompanied with sudden cardiac death [1]. This disease is mainly detected in developed countries, but it also affects the developing countries. It is considered the main cause of death worldwide [2]. Furthermore, MI survivors are susceptible to a high risk of heart failure (HF) [3]. The MI-related HF causes high morbidity and mortality rates. It affects around 6 million people in the United States with 300,000 incidences of death per year costing approximately $40 billion representing a huge burden on the healthcare system [4].

MI, also known as heart attack, is defined as the complete blockage of heart microcirculation leading to hypoxia and death of cardiomyocytes. This blockage is caused by the narrowing of blood vessels where the heart does not receive enough oxygen (ischemia) leading to the disturbance of proper blood supply to different organs in the body. It is worth mentioning that atherosclerosis, which is the plaque formation, is the main cause of ischemia [5].

There are several types of MI depending on its pathophysiology. Type 1 MI is due to an atherosclerotic plaque erosion or rupture causing partial or complete blockage in myocardial blood supply leading to myocyte necrosis. Type II MI is also a spontaneous event; however, the underlying cause is not a coronary artery disease. It is mainly caused by an imbalance between oxygen supply and demand due to various reasons as coronary artery spasm, HF, anemia, hypotension, hypertension, renal failure, or major procedures [6]. Type III MI results in sudden unexpected cardiac death even before any increase in biomarkers levels could be detected [7]. Moreover, type IV and V are due to surgical interventions as percutaneous coronary intervention (PCI), stent thrombosis and coronary artery bypass grafting (CABG).

**Fig. 1:**
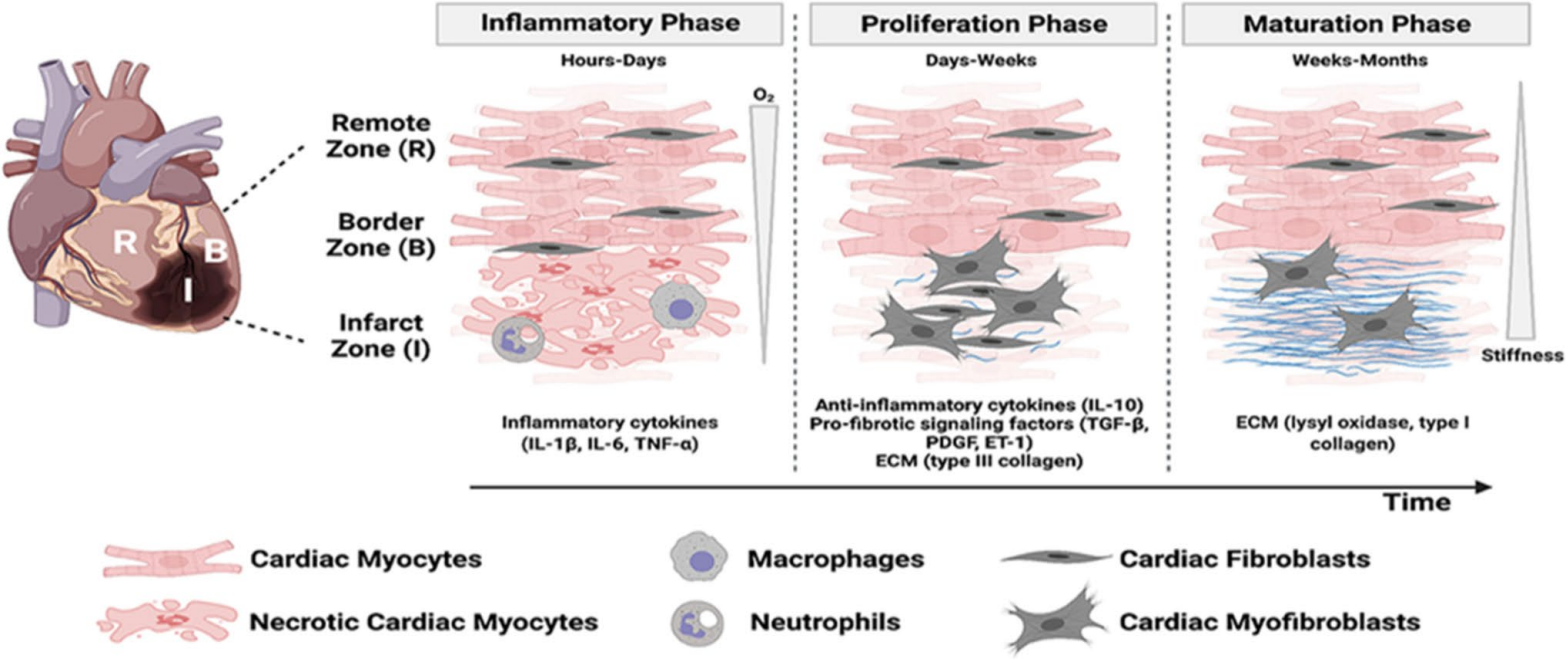
Illustration of the infarction microenvironment and the three healing phases [8]

It is worth mentioning that following the incidence of MI in human patients, more than one billion cardiac cells are being lost by necrosis and less often by apoptosis. Knowing the fact that the adult mammalian heart has negligible regenerative capacity, during the healing phase, the dead cardiomyocytes are replaced with non-contractile fibrotic scar to maintain the cardiac integrity avoiding any catastrophic events as cardiac rupture. The infarct healing process could be then divided into three different phases: inflammatory, proliferative/fibrotic, and maturation/ventricular remodeling phase as shown in Fig. 1.

The acute inflammatory phase lasts from 1–7 days during which the necrotic cardiomyocytes activate several immunological pathways [9]. The surviving cardiac cells and resident fibroblasts trigger inflammation by activating several pro-inflammatory signaling pathways. Subsequently, the neutrophils and monocytes are recruited to the infarcted region to remove dead cells. Moreover, pro-inflammatory macrophages are also recruited to the injured area and they secrete pro-inflammatory cytokines and matrix metalloproteinases (MMPs) causing phagocytosis and tissue digestion [10]. As a result, the extracellular matrix is altered due to degradation of collagen and hyalournan by MMPs followed by matrix deposition of fibrin providing the needed structural support for infiltration of immune cells. Despite the importance of early inflammatory activation to promote the reparative program, an excessive prolonged inflammatory phase can lead to adverse tissue damage, extensive cell loss, defective scar formation and consequently, infarction expansion [11].

The proliferative phase extends from 1–3 weeks. It is mainly characterized by removal of apoptotic neutrophils and activation of an anti-inflammatory pathway to allow the heart to repair itself. Anti-inflammatory macrophages, leukocytes and lymphocytes are involved in this phase by contributing to the alleviation of inflammation and promoting angiogenesis [10]. This phase is also characterized by cardiac fibroblast proliferation and migration to defected myocardium. Cardiac fibroblasts thus become the most abundant cell population in the heart during this stage, where they differentiate into activated myofibroblast phenotype. These cardiac myofibroblasts play an important role in deposition of collagen and fibronectin in addition to synthesis of other extracellular matrix (ECM) proteins that provide the structural support, maintain tissue integrity and contribute to scar formation. These cells also secrete anti-inflammatory molecules and pro-angiogenic factors to promote the reparative process [12]. The goal of this stage mainly is to replace the necrotic cardiac tissue with viable fibrotic tissue to fulfil the needed physical support. On the other hand, the excessive deposition of ECM proteins will exagerate the fibrosis process attenuating the heart contractility and increasing the risk of heart failure [13].

Finally the third stage; the scar maturation once initiated, lasts from weeks to months. During this phase, the composition of ECM is altered through crosslinking of collagen to provide myocardial strength. Additionally, the number of cardiac fibroblast decline by becoming quiescent and undergoing apoptosis. Moreover, angiogenesis in this stage is terminated [14]. Unfortunately, the formation of non-functioning fibrotic tissue during the healing process, represents a burden on the distant viable left ventricular region in order to maintain an adequate cardiac output. This pressure and load can reactivate the inflammatory and fibrotic pathways causing extensive left ventricular remodeling. Moreover, the persistance of cardiac fibroblasts within myocardium can extend up to years after the injury. Their presence can promote fibrosis in non-infacted myocardial regions leading to heart failure [15].

## Conventional management of MI

Conventional treatement of MI mainly relies on rapid restoration of cardiac blood flow using pharmacological and catheter based interventions as well as reducing the risk of any recurrent coronary thrombotic events [16].

The administration of thrombolytic therapy within the first 6h from MI incidence can be the most effective curative therapy to restore perfusion [17]. They act mainly by dissolving the blood clot. However, if the main cause of thrombus is not known and addressed, the patient may face the risk of re-thrombosis incidence within the first hours of MI [18]. Additionally, these thrombolytic agents are associated with some complications as spontaneous hemorrhage [19], hypotension, allergic or anaphylactic reactions [18]. Antiplatelet agents as aspirin or antithrombin agents as heparin are also used to restore blood flow.

Due to limitations of thrombolytic agents, percutaneous coronary intervention (PCI) has been used for perfusion. It is a non-surgical procedure in which a guide catheter is introduced into blocked coronary artery to inject a contrast dye and visualize the blockage. Simultaneously, another catheter with a balloon at its tip is inflated to unclog the blockage and restore vascularization. The implementation of a stent may be also required to maintain the coronary artery patency for a longer period of time [20].

Coronary artery bypass grafting surgery (CABG) is an effective surgical intervention to restore blood flow. A healthy vessel is grafted within diseased coronary artery to bypass the blocked region [21]. CABG is a practical solution to avoid incidence of restenosis, in addition, it can minimize hospital mortality. However, it is an expensive procedure with the possible incidence of post-surgical complications.

Nevertheless, it has been proven that the restoration of blood flow either by PCI or thrombolytic therapy during ischemia causes an injury called “ Ischemia/reperfusion injury (I/R)”. Numerous studies have investigated the underlying causes of I/R which include oxidative stress, apoptosis and other interrelated mechanisms that aggravate cardiomyocytes death [22].

Thus, the main treatment goals following immediate reperfusion include stabilization of coronary lesion, treatment of ischemia and secondary prevention.Traditional pharmacotherapy used to treat MI patients include β-blockers and nitrates. β-blockers reduce the load on myocardium by lowering blood pressure and heart rate. They exert anti-inflammatory, anti-apoptotic and antifibrotic features, however, their effect is restricted due to poor bioavailability, limited permeability, short half life and their adverse reactions [23, 24]. It is worthmentioning that after stabilization of patient’s condition, several cardioprotective drugs and life style modifications should be done to prevent long term complications. Cardioprotective drugs in secondary management aim to reduce elevated blood pressure and monitor cholesterol levels using several drugs as angiotensin converting enzymes inhibitors (ACEIs) and statins [25]. Concerning ACEIs, they decrease blood pressure, reduce ventricular remodelling and minimize cardiac hypertrophy, thus reducing mortality rate. However, they cause several side effects as angioedema, hypotension and renal failure. Statins act by preventing plaque formation through the reduction of low denisty lipoprotein (LDL) in addition to the attenuation of inflammation [26]. On the other hand, statins are characterized by low bioavailability and extensive first pass metabolism. Moreover, they increase risk of myopathy. Thus, the use of both ACEIs and statins are limited due to their aforementioned drawbacks.

## Novel therapeutics approaches to promote cardiac regeneration

The current pharmacological and surgical therapies managed to reduce mortality rate, but they failed to address and compensate the loss of cardiomyocytes and vasculature [27]. Therefore, the future therapeutic target should focus on cardiac regeneration to restore myocardial contractility and reduce incidence of MI-HF. The recent approaches that can enhance the regeneration of myocardium are mainly focused on cell-based therapies [28] and cell-free approaches.

### Cellular based cardiac regeneration approaches

Initially, due to inability to obtain functional cardiomyocytes to restore myocardial contractility, non-cardiac cells were transplanted as skeletal myoblast. The skeletal myoblasts were extracted from skeletal muscles of patient, expanded and implanted within heart. However, it did not improve contractility of heart and caused several complications [29]. The more recent cellular generations used human mesenchymal stem cells (hMSCs) that were purified from bone marrow aspirate. Adipose tissue also represented another source for purification of hMSCs. These cells showed a trend towards improvement of cardiac function and increased vasculature in the infarct [30]. Due to doubt in the cardiomyogenic potential of MSCs shown in some studies, there has been a shift towards cardiac stem cells (CSCs). These are resident cardiac cells with self-renewal potential. Limited clinical data investigated the implementation of CSCs and reported its low engrafment potential. In addition, it did not show an improvement in ventricular function and was not considered as an effective therapy for treatment of heart failure [31]. Finally, the interest was shifted towards pluripotent stem cells to overcome the doubt of the ability of MSCs to differentiate into cardiomyocytes. The pluripotent cells can differentiate into cardiomyocytes in vitro before being transplanted within the heart [32].

#### Limitations of cell-based therapies

**Fig. 2:**
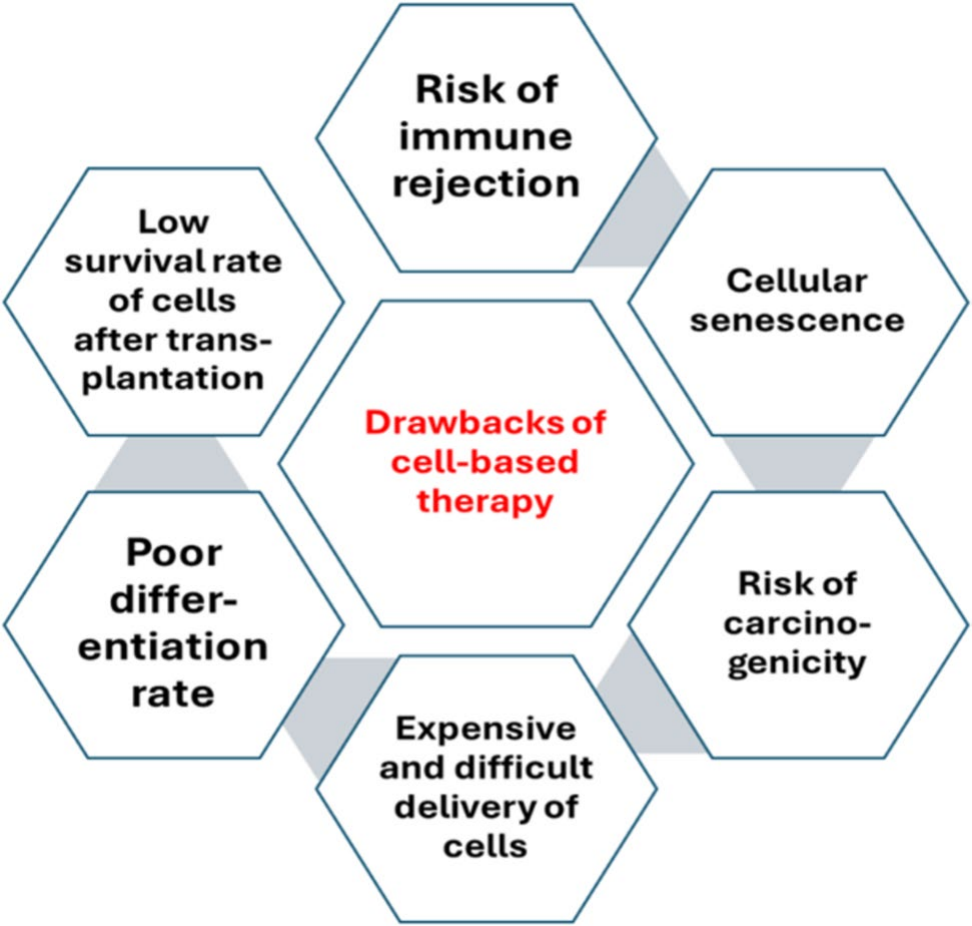
Drawbacks of cell-based therapy [34]

The cell based therapy faces a great challenge that constrains its efficacy mainly due to poor survival and low engrafment rate of transplanted cells as shown in Fig. 2. The rough ischemic environment due to injury limits the endurance of cells [33]. Moreover, obtaining the required number of cells is also challenging as source tissue does not contain enough cells. Therefore, the cells should be cultivated in vitro for expansion. However, the cells become more liable to aging with subsequent morphological and genetic changes that affect their proliferation and survival. In addition, senescent cells may affect the surrounding cells by spreading senescence through the secretion of certain factors which attenuate the success of the regeneration therapeutic approach.

The route of administration is another factor that affects engrafment rate of transplanted cells. Cells can be delivered by several routes including intramyocardial, intracoronary and intravenous route. The intramyocardial route is the most common route for cell delivery, however, it is considered as an invasive route that requires open chest surgery. Intracoronary injection is accomplished through injection of cells within the coronary artery or cardiac vein and it is less stressful than intramyocardial route. The intravenous route despite being the least invasive route, leads to major loss of transplanted cells due to entrapment of cells within different organs during their movement within blood stream. Moreover, the transplanted cells carry the risk of teratoma formation and risk of rejection.

Despite that some theories still attribute the beneficial effects of cellular therapy, there has been an increased interest in discovering new cell-free cardiac regeneration approaches due to the presence of major challenges facing the cell-based approach of cardiac regeneration such as low engrafment and survival rate to the paracrine hypothesis.

### Cell-free approaches to promote cardiac regeneration

An approach in cell- free therapy includes the use of secreted products from cells to act as the therapeutic material rather than the cell itself including cell secretomes and extracellular vesicles. A key element is the use of stem cells as they are considered as a key therapeutic tool in the field of regenerative medicine including cardiac regeneration. They secrete active molecules including various growth factors and cytokines [35] that protect the surrounding cardiovascular tissue by activating angiogeneic, antifibrotic and antiapoptotic paracrine pathways [36]. The use of exosomes as an example of extracellular vesicles has evolved as a cell-free approach to enhance cardiac regeneration. Exosomes are nano-sized vesicles secreted by cells containing bioactive molecules as protein, RNA, DNA and lipids. These exosomes can enhance angiogenesis, reduce fibrosis and improve ejection fraction of left ventricle [37].

Other alternative regenerative cell-free approaches include innovative in vivo reprogramming strategy that depends on conversion of cardiofibroblasts into cardiomyocyte like cells [38, 39].

Nanotechnology, on the other hand has offered a huge platform to adminster different drugs, growth factors, natural products that can reduce the size of infarction and promote cardiac regeneration. This can be achieved by different mechanisms including:

- Targeting excessive fibrotic response post MI to reduce cardiac remodelling [40].
- Scavenging the accumulated reactive oxygen species (ROS) that trigger DNA damage causing cellular senescence and death, which represents a great barrier towards cardiac regeneration [41].
- Attenuating excessive post MI inflammation that exagerates cardiomyocyte injury and threatens its survival. Attenuating the inflammation leads to formation of tensile healing scar through suppression of protease enzyme activity and the activation of certain chemokines that recruit progenitor cells to infarct site promoting angiogenesis and promoting cardiac regeneration [42].
- Administrating moieties with anti-apoptotic potential improving survival of resident cardiomyocytes.
- Enhancing angiogenesis and promoting perfusion within infarction, thus enhancing its recovery [43].

Nanomedicine can offer different nanocarriers to enable the delivery of various therapeutics to the ischemic myocardium performing the aforementioned mechanisms and promoting cardiac healing post MI. These nanocarriers have many advantages including the ability to target the therapeutics directly to the ischemic myocardium. In addition, the nanocarriers can protect therapeutics liable to degradation and control their release [44, 45]. Nanoformulations can be prepared using different materials including polymers ( as Polylactic co-glycolic acid “PLGA”), lipids and metals (as gold and silver). Nanomedicines targeting myocardium can be administered using different routes as intramyocardial, intracoronary and intravenous. The intramyocardial route is highly invasive and might cause further injury to weakened myocardium. The intracoronary route is less invasive, as it depends on catheter-based delivery of therapeutics directly to coronary artery [46]. However, it carries the risk of embolism in addition to that the catheter is not always compatible with all the cargo and the nanocarriers used. Therefore, the intravenous route remains the least invasive and preferred route for delivery. Nanomedicine targets the diseased organ either by passive or active targeting. Passive targeting is based on the physicochemical properties of the diseased organ and enhanced permeation and retention phenomenon (EPR), while active targeting relies on the utilization of certain ligands. This review will focus on nanocarriers loaded with cell-free therapeutic as drugs, natural products, genetic material or growth factor that is passively or actively targeted to ischemic myocardium to promote its regeneration.

#### Passive targeting

The enhanced permeation and retention ( EPR) effect was reported in the myocardium after infarction and ischemia [47]. EPR means that there is preferential accumulation of nanocarrier in diseased organ after being extravasted from blood due to presence of abnormal highly permeable vasculature within this organ due to injury or inflammation. This phenomenon was first described in cancer as rapid proliferation of tumors requires increased oxygen demand promoting angiogenesis. However, the formed vessels become chaotic and leaky [48]. The EPR effect also occurs after myocardial infarction due to upregulation of vascular endothelial growth factor (VEGF) that tends to increase vascular permeability and promotes angiogenesis within ischemic myocardium to aid in restoring blood flow and oxygen supply [49]. However, the post MI-EPR effect is characterized by being poor and tends to disappear within 48 h unlike that of cancerous tissue. Despite this limitation, several studies have reported that nanocarriers whose size ranges between 100–200 nm are advantageous through post MI-EPR effect [50, 51].

Polymeric nanocarriers are characterized by being stable, where they can protect encapsulated cargo and offer a sustained drug release manner. A sustained release pattern is required to provide maximum protection of cardiomyocytes and promote cardiac regeneration [52]. Polylactic co-glycolic acid (PLGA) is a biodegradable and biocompatible polymer that has been approved by food and drug administration (FDA) [53] and extensively used in targeting the ischemic myocardium.

Oduk et al. encapsulated VEGF within PLGA NPs (113 nm) to provide a sustained release manner for at least 31 days. A low dose of VEGF was used to avoid its associated side effects as hypotension, retinopathy or any other safety concerns. Using a murine myocardial infarction model, the nanoparticles were injected into the peri-infarct region where the VEGF promoted revascularization of ischemic myocardium, reduced the size of infarct and improved left ventricular contractility. Thus, this was considered as a novel approach for cardiac regeneration [44, 45]. Mao et al. also formulated PLGA NPs loaded with pitavastatin to target inflammatory cells within ischemic myocardium. The delivered drug coordinated the recruitment and mobilization of monocyte/macrophage to the diseased myocardium. Thus, it reduced the excessive prolonged inflammation that exagerates the fibrosis leading to left ventricular dilation and thinning subsequently, causing heart failure. Therefore, it was considered as a clinically feasible nanosystem to reduce post infarction fibrosis and decrease incidence of poor prognostic heart failure [54].

Some research results have demonstrated the ability of some natural products to promote the inherited mechanism of cardiac regeneration through enhancing angiogenesis, preventing cardiomyocyte apoptosis besides increasing the migration, proliferation and differentiation rate of progenitor cells into cardiac ones [55]. The application of nanomedicine can minimize the limitations accompanied by use of natural products as poor solubility, limited bioavailability and rapid degradation rate. Nabofa et al. encapsulated two natural products;curcumin and nisin within biodegradable PLGA NPs. Curcumin is a polyphenol with cardioprotective potential attributed to antiinflammatory and antioxidant properties. Nisin is an antimicrobial peptide that was combined with curcumin to reduce cardiac related defects. The cardioprotective potential of formulated system was tested using isoproterenol-induced MI guinea pig model. The histological examination showed that the pretreatment of guinea pigs with CurNisNPs before induction of infarction prevented necrosis and increase in myofibril thickness. In addition, the oxidative stress markers were reduced, thus CurNisNPs increased the cardiac antioxidant defense [56].

Jing and Chen et al. selected Wogonin; a flavonoid with cardioprotective properties and loaded it within PLGA NPs. Using an isoproterenol-induced MI rat model, the formulation was given orally as a pretreatment for 21 days and several investigations were conducted to examine the cardioprotection efficacy. The treated rats showed a significant decrease in infarct size compared to untreated rats. In addition, edema and necrosis were reduced. Moreover, the levels of different cardiac proteins as Lactate dehydrogenase (LDH), Creatine kinase -MB (CK-MB) and cardiac troponin-T (cTn T) were decreased [57].

Bejerano et al. explored the possibility of using microRNA-based therapy to target post-infarction cardiac macrophage and switch their phenotype from inflammatory to reparative manner, thus promoting cardiac healing. They formulated polymeric NPs spontaneously formed due to the complexation of hyalournan -sulfate with miRNA-21 through calcium ion bridges yielding anionic particles with average size of 130 nm. This system promoted angiogenesis, decreased the rate of cardiomyoctes apoptosis and reduced left ventricular remodelling post-infarction [58].

Boarescu et al. also examined the cardioptotective potential of curcumin loaded polymeric nanoparticles using acute myocardial infarction model in diabetic rats. The curcumin was chosen due to its antioxidant, anti-inflammatory effects in addition to reduction of necrosis and apoptosis of cardiomyocytes [59]. Curcumin nanoparticles showed cardioprotective effects manifested by the prevention of cardiac enzymes elevation and oxidative stress markers [60, 61].

The metal nanoparticles have been also used in the treatment of various cardiovascular diseases due to their distinct electrical and visual properties. Their small size that ranges from 10 to 100 nm enables them to interact well with cells. Their cardioprotective effect has been reported through various mechanisms of action [62]. The metal NPs possess anti-inflammatory, anti-hypertrophic, anti-oxidant properties and protect against myocardial damage. Their ability to scavenge reactive oxygen species (ROS) reduces the oxidative stress. This oxidative stress damages the cells, therefore the ability of metal NPs to enter cells and reduce ROS, eventually decreases the apoptosis rate. The toxicity profile of metal NPs is dependent on many factors as size, shape, method of preparation and charge. More intense research is required to investigate the potential toxicity of these NPs. In addition, different NPs coatings can affect their toxicities [63].

Tian et al. formulated polyethylene glycol coated gold nanoparticles (AuNPs, 10 nm) to evaluate their effect in acute MI. AuNPs tend to accumulate within infarcted myocardium. The infarction model was induced using coronary ligation surgery and the nanoparticles were intravenously given for 7 days after recovery from surgery. The echocardiohraphy showed the ability of AuNPs to preserve left ventricular wall integrity and reduce its dilatation in addition to improving the systolic function. Moreover, the histological examination revealed a decrease in infact size in the treated groups. The AuNPs did not decrease the cardiomyocytes apoptosis rate, however they reduced the adverse cardiac fibrosis and decreased the excessive inflammation [64].

Lipid nanoparticles are biocompatible, affordable and can be easily formulated. They are good candidates for passive targeting by adjusting size, polarity and membrane rigidity. They have been extensively studied in the field of cancer treatment, and they represent promising candidates for tageting the infarcted heart [65].

Shouwen Zhang et al. formulated Baicalin loaded nanostructured lipid carriers (NLCs) and pegylated ones to passively target the infarcted myocardium. It was reported that Baicalin can reduce the MI through their antioxidant and antiapoptotic properties. The formulated nanosystems were accumulated within ischemic myocardium and significantly reduced the infarct size induced by coronary artery permanent ligation animal model [66].

Allijn et al. formulated pegylated liposomes (110 nm) loaded with Berberine. This natural product was selected due to its antiinflammatory, antioxidative and cardioprotective properties in addition to its ability in improving cardiac function. The pegylation aimed to increase circulation time of liposomes. Myocardial infarction was induced in mice by surgical permanent ligation of coronary artery. The formulations and free drug was administered intravenously. The distribution of liposomes was visualized and confirmed accumulation of liposomes within infarcted myocardium. The berberine liposomes significantly preserved left ventricle ejection fraction, decreased the deterioration in contractility, reduced hypertrophy and adverse cardiac remodeling. It enhanced cardiac function 64% compared to free berberine [67]. More recent research publications using passive targeting technology in targeting infarcted myocardium are included in Table 1.

**Table 1:** Recent research publications using passive targeting technology in targeting infarcted myocardium

Nanocarrier type	Target/rational for cardiac regeneration	API	cell line/animal model	Therapeutic effect	ref
PLGA NPs (113 nm) formulated using double emulsion method	The NPs enabled the local sustained release of VEGF to revascularize the ischemic myocardium	VEGF	Surgically induced MI using immunocompromised NOD/SCID mice	VEGF promoted revascularization of ischemic myocardium, reduced the size of infarct, and improved left ventricular contractility	[44, 45]
PLGA NPs	The rationale was the reduction of monocyte/macrophage recruitment to infarcted heart to minimize the post-MI excessive inflammation	Pita-vastatin	Permanent coronary ligation induced MI mice model	The echocardiography and histological analysis showed that injecting 1 mg/kg Pitavastatin-PLGA NPs for 3 consecutive days limited the negative LV remodeling and decreased the accumulation of monocytes within infarcted myocardium	[54]
Spontaneously assembled anionic NPs (130 nm) due to complexation between hyaluronan sulphate and nucleic acid by calcium ion bridges	Targeting post MI inflammation through the delivery of nucleic acid to cardiac macrophages that are upregulated within infarction zone to phagocytose the apoptotic cells	miRNA-21	Coronary ligation animal model	The intravenously injected formulation successfully increased the anti-inflammatory macrophages manifested by increased TGF and angiogenesis. Moreover, the echocardiography analysis showed a reduction in left ventricular dilatation and mass	[58]
Biodegradable polymeric NPs formulated using PLGA and PLA (284 nm)	The application of nanotechnology to improve stability and bioavailability of nutraceuticals as curcumin. Curcumin can exert anti-inflammatory and cardioprotective effects. In addition, it was combined with Nisin, an anti-microbial peptide, to treat cardiac related defects	Curcumin & Nisin	Isoproterenol induced acute MI in guinea pig	CurNisNp system exerted cardioprotective features as manifested in guinea pig myocardial infarction model. The administered system reduced the cardiac hypertrophy and histological analysis proved the prevention of necrosis	[56]
Cu was loaded within polymer-based NPs(30–100 nm) purchased from CVI Pharma (Vietnam)	The NPs can improve the bioavailability of Cu compared to crude drug. Thus, Cu NPs can exert better antioxidative effect and attenuate cardiac damage after myocardial ischemia	Cu	Isoproterenol induced MI animal model. Crude Cu and Cu NPs were administered orally for 15 days as a prophylactic pretreatment	Cu NPs prevented elevation in MI enzymes compared to crude curcumin In addition, Cu NPs showed better histopathological results after the administration of high dose of curcumin	[61]
PLGA NPs	The use of biodegradable NPs in the treatment of cardiovascular diseases to compare their effect relative to unformulated API	Resveratrol	Isoproterenol induced acute MI	Pretreatment with PLGA nanoparticles loaded with resveratrol showed superior protective effects compared to unformulated resveratrol as manifested by histopathological assessment and evaluation of enzymes besides inflammatory markers	[68]
Polymeric NPs (30–100 nm)	Cu nanoparticles could exhibit anti-inflammatory & antioxidative effects	Cu	Isoproterenol induced acute MI	The pretreatment protocol using CU NPs showed cardio-protective effects manifested by the prevention of cardiac enzymes elevation and oxidative stress markers	[60]
Polymeric nanoparticle (mPEG-PLA-TPGS NP) (100–200 nm)	The nano-formulation can improve the poor physical properties of Tanshinone II A and thus, improve adverse cardiac remodeling	Tanshinone II A	Coronary ligation animal model	The echocardiogram showed that tanshinone NPs reduced left ventricular dilatation and decreased attenuation in left ventricular ejection fraction. Moreover, NPs inhibited apoptotic cardiomyocyte death and adverse cardiac remodeling after MI	[69]
PLGA NPs (194.82 mn) that were formulated using PVA as a stabilizer	Formulating Wogonin within polymeric nanoparticles could improve its poor solubility and short retention time in the body	Wogonin	Isoproterenol induced acute myocardial infarction in Wistar rats	Pretreatment using Wogonin nanoparticles decreased the myocardial infarction magnitude proving its cardioprotective potential	[57]
Gold NPs (50 nm)	Accumulation of gold nanoparticles within endothelial cells and capillaries of infarcted myocardium	……	Isoproterenol induced acute MI in adult male albino rats	Gold NPs protected the normal architecture of cardiomyocytes from the damage. This was manifested using the histological examination and biochemical measurement of cardiac enzymes	[70]
Alginate stabilized Ag NPs (8.72–37.84 nm)	The use of antioxidant and anti-inflammatory effects of silver nanoparticles in the management of MI	………	Isoproterenol induced acute MI	The Ag NPs did not cause any kidney or liver damage. It showed cardioprotective effect against isoproterenol MI model manifested by preventing the elevation of CK-MB and LDH levels besides histopathological examination	[71]
PEG-coated AuNPs (10 nm)	The choice of AuNPs was attributed to their ability to aggregate within infarcted myocardium	……….	Coronary ligation animal model. The treatment was given IV by tail injection for 7 days after recovery from surgery	The AuNPs decreased the infarct size as manifested by histological examination. This was attributed to the potential of AuNPs in alleviating excessive inflammation and reducing cardiac fibrosis	[64]
NLCs and pegylated NLCs (83.9 nm)	The tendency of Pegylated NLCs to accumulate within infarcted myocardium attributed to EPR effect	Baicalin	Acute MI model was achieved by the permanent ligation of left coronary artery method	The Baicalin-NLCs and pegylated NLCs resulted in a significant reduction in infarct size compared to Baicalin solution and untreated group	[66]
Liposomes (110 nm) prepared by ethanol injection method	The ability of liposomes to extravasate through EPR effect and accumulate within infarcted myocardium as confirmed by distribution study	Berberine	MI was induced in mice by permanent ligation of left anterior descending artery	The Berberine-liposomes preserved left ventricle ejection fraction compared to free berberine and blank liposomes. In addition, they decreased the deterioration in cardiac contractility, hypertrophy of left ventricle and reduced cardiac adverse remodeling	[67]

Nanoparticles, NPs-Curcumin, CU-Polylactic acid, PLA-Poly lactic co glycolic acid, PLGA- Myocardial infarction, MI-monomethoxy-poly (ethylene glycol)-poly (lactic acid)-D-a-Tocopheryl polyethylene glycol 1000 succinate, mPEG PLA TPGS- Polyvinylalcohol,PVA- silver nanoparticles, Ag NPs- gold nanoparticles, AuNPs- nanostructured lipid carriers, NLCs- enhanced permeation and retention, EPR

#### Active targeting

**Fig. 3:**
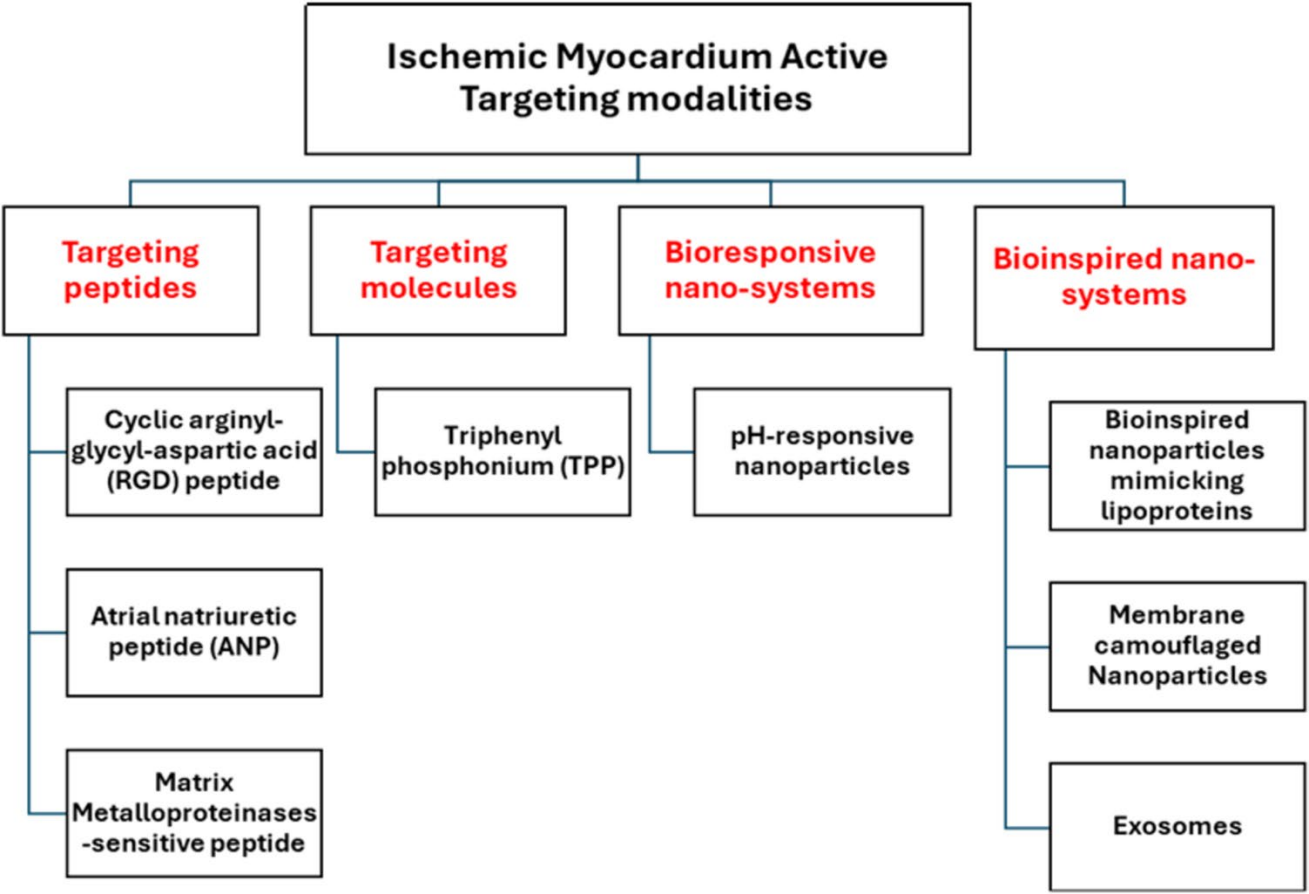
Modalities to actively target the ischemic myocardium

Active targeting refers to decorating the nano-carriers with certain ligands to seek an overexpressed target within diseased organs compared to the rest of the body. Various ligands were used to target the ischemic myocardium as illustrated in Fig. 3.

##### Targeting peptides

Angiogenesis is a crucial process during the healing of ischemic myocardium and is characterized by overexpression of signalling molecules as integrins. αvβ3 integrin is a cell membrane glycoprotein receptor that is activated and upregulated on endothelial cells during angiogenesis occuring as a part of infarction healing [72]. Cyclic arginyl-glycyl-aspartic acid (RGD) is a peptide that has good affinity to αvβ3 integrin receptors, thus it can be used as targeting peptide to deliver cardioprotective cargo to the infarction.

Zhaoqiang Dong et al. formulated pegylated SLNs coated with RGD peptide for treating MI. Puerarin was the selected therapeutic agent for its antioxodant and ROS scavenging capabilities. The formulated system that was decorated with RGD showed a preferential accumulation within ischemic myocardium. In addition, it achieved the best therapeutic outcome by regarding the infarct size compared to free drug [73].

Atrial natriuretic peptide (ANP) is a cardiac hormone that regulates blood pressure by stimulating vasodilation in addition to its anti-hypertrophic properties [74]. It can be used as a targeting ligand to the infarcted heart where its receptors are overexpressed, however they are also present in other tissues as kidneys, lungs, brain, testes, adipose tissue, adrenal gland tissues and vascular smooth muscle cells [75]. In order to potentiate the targeting capability of ANP, it is sometimes combined with triphenylphenylphosphonium (TPP). TPP cations can cross passively the lipid bilayer and accumulate within mitochondria due to its high negative membrane potential [76].

Jie Wang et al. decorated a Baicalin lipid-polymer nanoparticle with dual ligands; ANP and TPP. The LPN sustained the release of Baicalin and the decorated system exhibited a slower release manner due to presence of ligands. The ANP/TPP LPN system did not induce any cytotoxicity upon human cardiac myocytes (HCM). A biodistribution study illustrated that ANP/TPP-BN-LPNs showed the highest accumulation within heart compared to single ligand nanoparticle, unmodified system (LPN) and drug solution. Moreover, it fulfilled the maximum reduction in infarct size and achieved the best therapeutic outcome compared to other nanoparticles and drug solution [77].

Matrix Metalloproteinases (MMPs) play an important role in degradation of extracellular matrix post-MI as they are involved in cardiac remodelling. Thus, multiple MMPs are elevated after the infarction. This enables studying MMPs as a potential therapeutic agent and can be utilized in the active targeting of ischemic myocardium [78].

Mingfeng Shao et al. formulated pegylated SLNs decorated with MMP sensitive peptide and loaded with a natural product “Schisandrin B” for its cardioprotective and antioxidant potential. An in vivo biodistribution study demonstrated the preferential accumulation of MMP-SLNs in the heart after 48 h compared to the drug solution. Moreover, this system exihibited the most significant reduction in infarct size achieved using an in vivo MI model induced by partial ligation of coronary artery [79].

##### Bio-responsive nanosystems as pH-responsive nanoparticles

Bioresponsive nanoparticles were formulated to hydrolyze or degrade in response to a change in pH, redox potential, reactive oxygen species or enzymes at diseased sites to guarantee preferential release of cargo at target site [80]. The cardiac ischemia induces the local acidification of environment, therefore a pH responsive nanosystem can be used to target MI.

Ling Wang et al. loaded an anti-inflammatory drug “Colchicine” within calcium carbonate nanoparticles. These calcium nanoparticles were pH-responsive that selectively released the drug at acidic inflammation sites. Using an in vivo MI animal model, the colchicine calcium nanoparticles that were injected into caudal vein after ligation of coronary artery achieved a better reduction in infarct size compared to drug solution. In addition, colchicine calcium nanoparticles reduced several inflammatory cytokines as TNF-α and CRP that are elevated during MI [81].

##### Bioinspired nanosystems

**Bioinspired nanoparticles mimicking lipoproteins** Formulation of biomimetic nanoparticles that resemble naturally occuring nanoparticles as lipoproteins can be considered as a promising approach for targeting cardiovascular disorders as MI. Lipoproteins are endogenous carriers of fats and cholesterol within the blood stream. These fats and lipids are hydrophobic, forming complexes with proteins called lipoprotein to facilitate their transportation through circulation. These lipoproteins are classified into various classes based on their size and denisty; chylomicrons, very low denisty protein (vLDL), low denisty lipoprotein (LDL) and high denisty lipoprotein (HDL). Chylomicrons and vLDL transport triglycerides while LDL and HDL transport cholesterol where LDL transports cholesterol from liver to different body tissues, while HDL transports cholesterol from body tissues to liver. LDL and HDL have emerged out as promising drug delivery vehicles. LDL is composed of an internal core of cholestryl esters and triglycerides surrounded by an outer monolayer of phospholipid decorated with apoprotein B (apoB). The apoproteins are responsible for receptor recognition, where LDL particles are recognized by LDL receptors (LDLR). HDL is also composed of a hydrophobic core composed of cholesterol ester and triglycerides coated with a hydrophilic layer of phospholipid and apoprotein A1 [82]. HDL is recognized by certain receptors as Scavenger receptor B1 (SR-B1) and ATP Binding Cassette Type A and Class 1 (ABCA1). HDL is commonly known as good cholesterol, it possesses anti-inflammatory and anti-oxidative characteristics [83].

In some neoplastic disorders, the increased need for cholesterol required by rapidly dividing cells leads to the overexpression of lipoprotein receptors as LDLR and SR-B1. Similarily after a MI episode, the demand for cholesterol increases to synthesize new cell membrane for newly formed scar tissue or myocardial fibers implicating an overexpression in lipoprotein receptors. de Lima et al. [84] reported that repairing the cardiac dysfunction and inflammation is accompanied by overexpression of LDLR. In addition, SR-B1 is also expressed upon several inflammatory and proliferative cells that are recrutied to the infarcted area as monocytes, leukocytes [85] and macrophages. Therefore, utilizing LDL and HDL as transport vehicles can be promising to target and repair the infarcted myocardium.

The isolation of natural LDL and HDL from fresh donor plasma is expensive, laborious and problematic. The obtained amount is limited, in addition the samples vary from batch to batch. Moreover, the isolated lipoproteins are only valid for limited periods before being subjected to aggregation and degradation. Therefore, various studies and attempts aim to prepare synthetic LDL and HDL-mimicking particles. They are mainly composed of phospholipid/cholesterol esters microemulsion prepared by certain ratios in addition to the appropriate apoprotein. However, obtaining the apoprotein from the donor plasma is also problematic owing to its size and complex nature. Alternative approaches have been developed to replace the use of natural apoproteins by synthesizing artificial apoprotein-like peptides [86]. Moreover, another approach was studied that mainly depends on formulation of protein-free microemulsion (LDE) whose lipid phase resembles that of native LDL. Upon injection, these particles acquire several exchangeable apoproteins from the circulation allowing their binding to lipoprotein receptors [87, 88].

Raul c Maranhão et al. reported the formulation of LDE whose composition resembles that of LDL loaded with methotrexate (MTX). It was postulated that the prepared system can acquire Apo-E and interact with LDLR. An in vivo experiment was carried out in which the MI was induced by occlusion of left coronary artery. The LDE-MTX decreased the infarction size and improved the left ventricle systolic function compared to the limited effect of crude unloaded MTX [89].

Richart et al. extracted human Apo AI from donor plasma which was utilized to form a complex with phosphatidylcholine to mimic the natural HDL particles. An in vivo study using a myocardial infarction animal model induced through surgical occlusion of left coronary artery illustrated the preferential accumulation of apo AI nanoparticles within ischemic tissue. In addition, the apo AI nanoparticles reduced the infarct size due to its antiinflammatory and immunomodulatory effect [85].

Exosomes (30–150 nm) have attracted attention as cell free bioinspired approach in cardiac regeneration. Exosomes can be derived from human or plant origin, however the human derived exosomes are more established in terms of characterization and analysis. Stem cells derived exosomes have been extensively studied for myocardial repair due to angiogenic and antiapoptotic properties [90]. They are considered as innate carriers that can deliver small molecules as proteins, nucleic acids and drugs. They exert a paracrine effect on infarcted myocardium and play an important role in its protection [91].

Peisen Huang et al. isolated exosomes from control mesenchymal stem cells(MSCs) and atorvastatin pretreated MSCs. It was reported that combining the MSCs with lipid lowering drug as atorvastatin can improve cardiac function for patients with coronary diseases. Using a surgically induced MI model, exosomes were intramyocardially injected into border zone of infarcted heart. Exosomes derived from atorvastatin pretreated MSCs significantly improved systolic function and decreased scar size compared to control exosomes and untreated animals. In addition, they enhanced angiogenesis and suppressed fibrosis. The atorvastatin treatment enhanced beneficial effects of control exosomes [92].

**Fig. 4:**
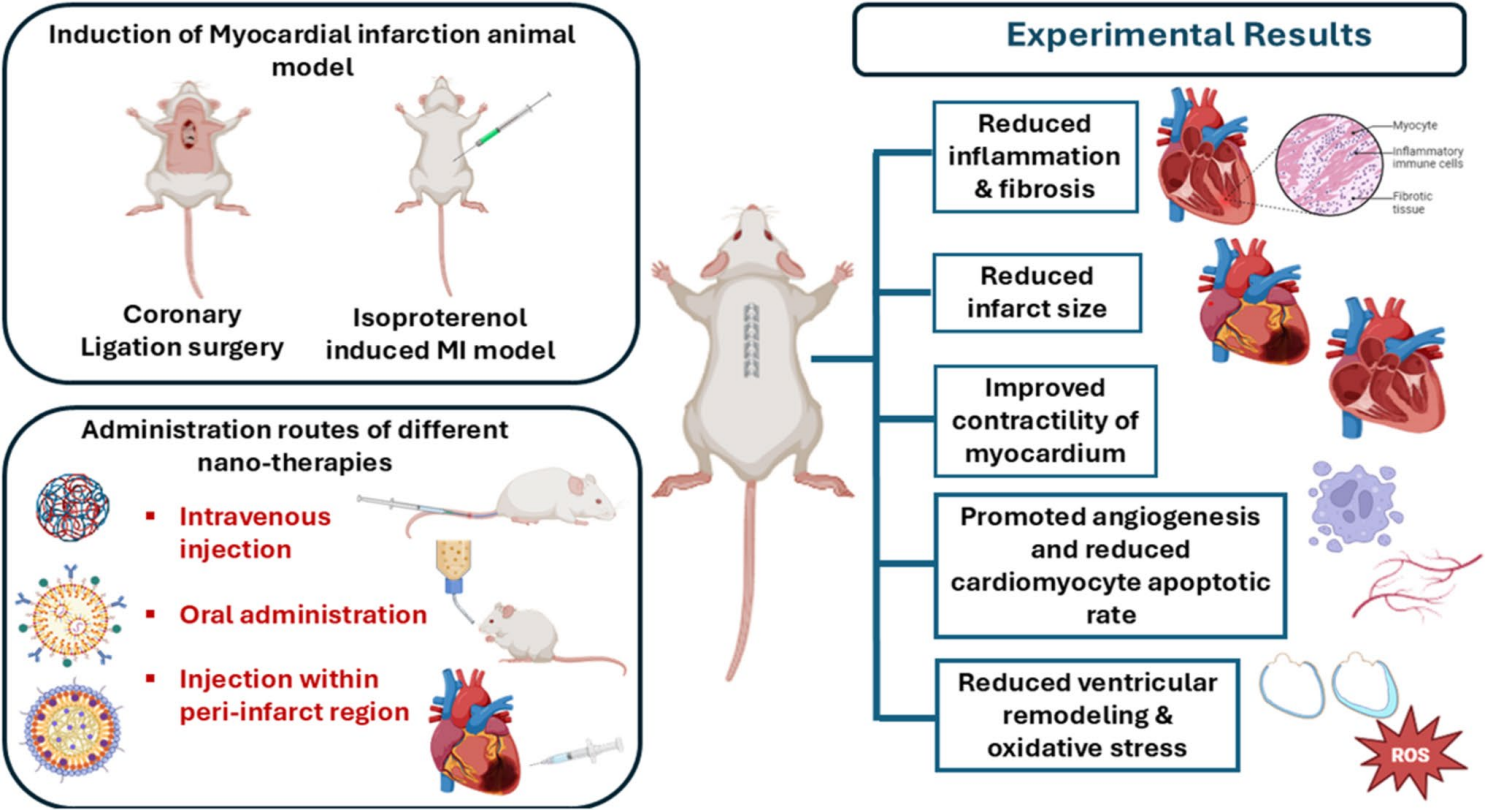
Schematic representation summarizing the main key results that indicate cardiac repair after administration of cell-free nano-therapies in a MI-induced animal model

The reported MI induced animal models and various routes of administration of cell-free nanotherapies that can enhance cardiac regeneration are summarized in a schematic Fig. 4 illustrating the key results that support the cardiac repair post infarction.

##### Membrane camouflaged nanoparticles

**Fig. 5:**
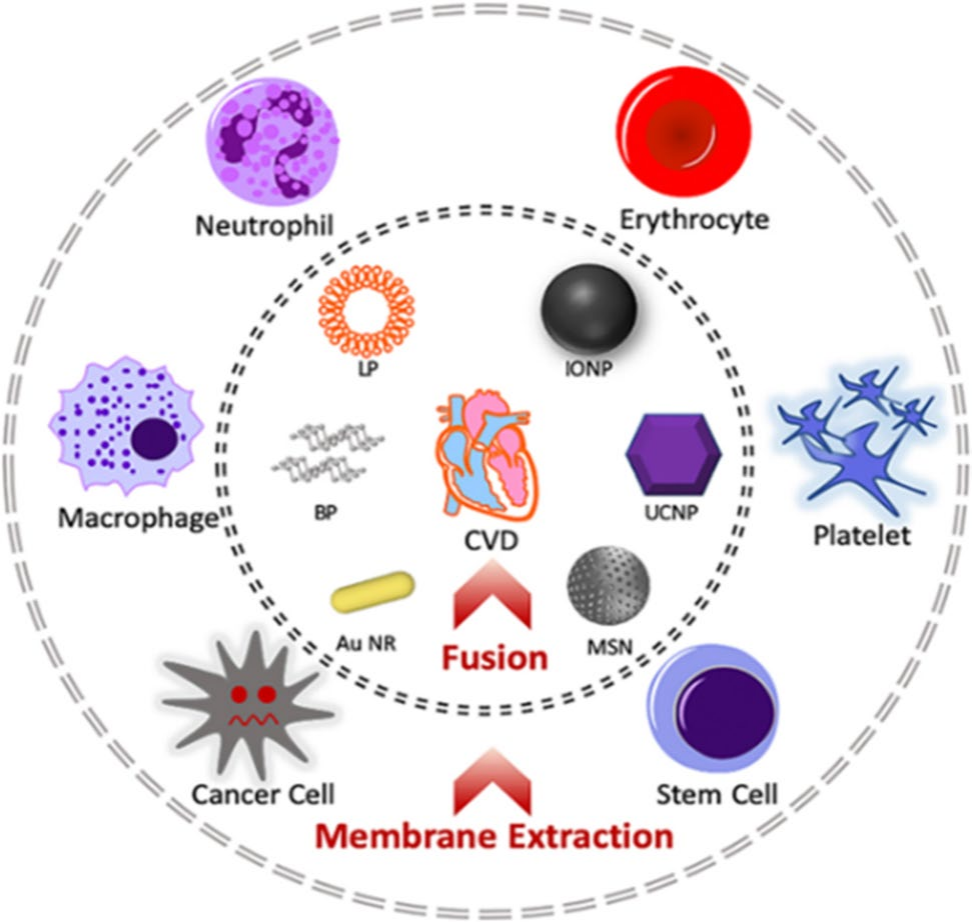
Illustration of the possibility of cell membrane extraction of different cells to be fused with various nanosystems [93]

Cell membrane coating technology utilizes an innate seperated cell membrane to coat a synthetic nanoparticle. This natural coat can aid to disguise the formulated nanoparticle from body`s immune clearance, thus increasing its circulation time. In addition, it is postulated that the cell-membrane coated nanosystem can inherit some intrinsic cell properties as specific targeting to a certain ischemic or inflammatory site and binding affinity to certain receptor or cell. Various cells are used for isolation of their membranes to coat different nanosystems as shown in Fig. 5 [93].

Dongjian Han et al. formulated polymeric nanoparticle composed of PLGA loaded with Interleukin-5 and coated with naturally derived neutrophil membrane. The formulated system promoted cardiac regeneration through enhancing angiogenesis. In addition, interleukin-5 promotes proliferation of endothelial cells besides increasing the recruitment of eosinophils to the injured heart that can repair injured tissue. The neutrophil coated nanoparticle was targeted to the ischemic myocardium owing to the long circulation time and chemotaxis targeting potential. It achieved the best therapeutic outcome after an in vivo induced MI experiment manifested by the highest reduction in infarct size and improvement in left ventricular ejection fraction [43]. More recent research publications using active targeting technology in targeting infarcted myocardium are included in Table 2.

**Table 2:** Recent research publications using active targeting technology in targeting infarcted myocardium

Nanocarrier type	Target/rational for cardiac regeneration	API	cell line/animal model	Therapeutic effect	Ref
RGD modified and pegylated SLNs loaded with Puerarin (110 nm)	RGD peptide can selectively bind to α_v_β_3_ integrin receptors, which are overexpressed on endothelial cells during angiogenesis	Puerarin	A cytotoxicity study was done using HCM. Acute MI model was induced by coronary artery ligation rat model	Using HCM cell lines, the free drug and formulated systems did not exhibit any cytotoxicity. The RGD-SLN resulted in the best treatment attributed to achieving the smallest infarct size	[73]
LPN composed of DSPE and PEG (75 nm)	The nanosystem was decorated with dual ligands; ANP and TPP to target ischemic myocardium	Baicalin	A cytotoxicity study was done using HCM. Acute MI model was induced by coronary artery ligation rat model	The nanosystem did not show significant cytotoxicity at the used drug concentrations. The ANP/ TPP-BN-LPNs achieved the highest reduction in infarct size	[77]
AcDEX NPs (pH responsive system)	The NP was modified by 2 peptides: ANP and TT1 peptides ANP allows accumulation of NPs within the infarcted heart TT1 peptide binds to a certain protein expressed on macrophages recruited within atherosclerotic plaque	two hydrophobic compounds, CHIR99021 and SB203580	Macrophage cell lines, RAW 264.7 and KG-1 in addition to primary macrophages of human and murine origin	AcDEX NPs exhibited preferential uptake with M2-like macrophages (approximately twofold and sixfold increase) in murine and human primary macrophages	[94]
SLNs conjugated with PEG and MMPs targeting peptide	Coating the nanoparticles with PEG could improve its circulation time. In addition, MMP sensitive peptide could actively target the upregulated proteolytic enzymes during MI	Schisandrin B (Sch B)	Partial ligation of coronary artery was used to induce MI rat model	MMP-Sch B SLNs could successfully target the heart and reduce infarction size. Therefore, it could be used for treatment of MI	[79]
LPNs were formulated and modified with TPP- TPGS	TPP cations can cross passively the lipid bilayer and accumulate within mitochondria due to its high negative membrane potential. The amphiphilic nature of TPGS can prolong circulation time, enhance the solubility of hydrophobic drugs, and improve cellular uptake	Tanshinone IIA (TN)	Primary cardiac cells and MI animal model induced through surgical occlusion of left coronary artery	The primary cardiac cells treated with TPP-TPGS-modified LPNs showed the highest cellular uptake. TPP-TPGS/TN/LPNs illustrated the highest accumulation within heart and highest reduction in infarct size	[95]
Calcium carbonate nanoparticles (243 nm) that were prepared using vapor-diffusion process	Calcium carbonate nanoparticles were characterized by being biocompatible and biodegradable in addition they were pH-responsive particles and could release their cargo at lower pH inflammatory sites	Colchicine	Surgical induction of myocardial infarction in a rat model	Colchicine loaded nanoparticles reduced the infarct size and myocardial fibrosis	[81]
Lipid core nanoparticles (60 nm) resembling the structure of low-density lipoprotein (LDL)	The formulated nanoparticles can acquire apolipoprotein E and accumulate within infarcted myocardium that overexpress LDL receptors	Methotrexate	MI animal model was induced through surgical occlusion of left coronary artery	The administered nanoparticles loaded with methotrexate reduced the infarction size and prevented post MI negative cardiac remodeling	[89]
n-Apo AI nanoparticles (human apolipoprotein AI which was purified from plasma and reconstituted with phosphatidylcholine). These nanoparticles resembled nascent HDL	N-apo AI would target the ischemic myocardium through preferential binding of the n-apo AI nanoparticles to recruited neutrophils through scavenger receptor BI	……	MI surgery was done through ligation of coronary artery for 30 min and then was released to simulate the reperfusion	n-Apo AI nanoparticles reduced the infarct size through its post ischemic anti-inflammatory and immunomodulatory effect	[85]
polymeric nanoparticle composed of PLGA coated with naturally derived neutrophil membrane	Neutrophil coating facilitated the adherence of system to inflamed endothelial cells via upregulated ICAM-1, an integrin ligand. In addition, camouflaged nanoparticles exhibited longer circulation time and preferential accumulation within heart through neutrophil chemotaxis targeting	Interleukin (IL) − 5	Mouse model of acute MI induced by ligation of anterior descending coronary artery	The neutrophil coated polymeric nano-system significantly reduced the infarct size, decreased the apoptotic rate of cardiomyocytes, and improved left ventricular ejection fraction. The coated NPs achieved better results than uncoated ones	[43]

### The use of biomaterial-based nanocomposites for cardiac regeneration and repair

**Fig. 6:**
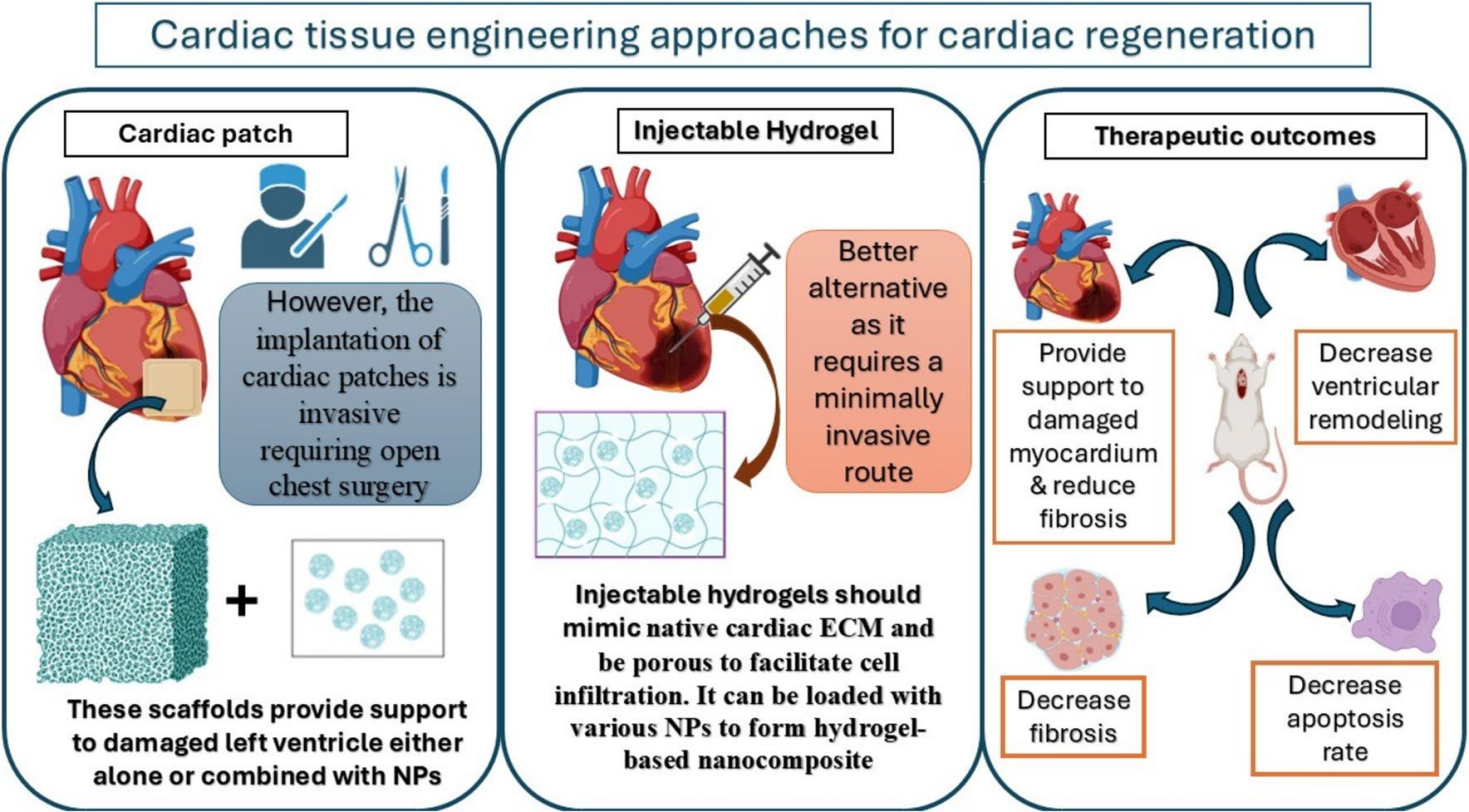
Schematic representation illustrating use of biomaterial-based nanocomposites for cardiac regeneration and summarizing main experimental results deduced from MI induced animal models

The ability of biopolymers to form an extracellular like matrix can be successfully utilized to improve cardiac regeneration post MI. These scaffolds can provide support to the damaged left ventricular wall of infarcted myocardium and improve the remodelling process [96, 97]. The cardiac tissue engineering approaches include combining the biomaterials with cells or more recently using the biomaterials alone to recruit cells to the damaged areas. Moreover, these biomaterials can be loaded with different growth factors, drugs, natural products and nanoparticles to potentiate the regenerative potential of the administered system. The biomaterials scaffolds are classified into cellular/acellular cardiac patches and injectable materials. The implantation of cardiac patches is invasive as it requires open chest surgery to be sutured to the epicardial surface of the heart. Therefore, injectable biomaterials represent a better alternative as it requires a minimally invasive administration route [98]. The cardiac tissue engineering approaches in cardiac regeneration field are illustrated in Fig. 6.

The ideal injectable biomaterials should preferably be biodegradable, biocompatible, mimicking native cardiac extracellular matrix and porous to facilitate cell infiltration. They can be either synthetic as poly(N-isopropyla crylamide) (PNIPAAM) and poly(ethylene glycol) (PEG) or naturally derived as fibrin, collagen, matrigel and chitosan. Recently, incorporation of various nanoparticles, nanotubes and nano-fibers within hydrogel was introduced to form hydrogel-based nano-composites. The addition of nano-formulations as gold nanoparticles and carbon nanotubes with injectable biomaterial enhanced angiogenesis, adhesion of recruited cells and electrical conductivity [99]. Moreover, combining nanoparticles and hydrogel can augment the therapeutic outcome. Rocker et al. reported the formulation of sulfonated gel incorporating VEGF, IL-10 and platelet derived growth factor loaded micelles. The formulated system showed a favourable sustained release of loaded cargo and significant increase in angiogenesis process [100].Moreover, nanobiomaterials such as nano-gels prepared from polymeric nanomaterial can be also used as an injectable biomaterial [101].Generally, the use of nanotechnology can enhance the physical properties of biomaterials augmenting its therapeutic effect. Thus, these injectable hydrogel-based nano-composites can be used as cell-free approaches to support the left ventricle, reduce negative remodelling and control the delivery of various cargos to ischemic myocardium.

Chen et al. formulated a new conductive hydrogel enriched with gold nanorods and nanoformulated Astragaloside IV(AST). The injectable hydrogel was composed of the hyperbranched poly (ethylene glycol) diacrylate/4-vinylphenylboronic acid (PEG-PBA) and thiolated hyaluronic acid (HA-SH). Upon injection, hydrogel exhibited good fluidity and was delivered via intramyocardial injection, then it was gelatinized *invivo* avoiding invasive surgeries. The gold nanorods improved conductivity of the system and promoted proliferation of cells. The AST is a natural product with anti-apoptotic and anti-inflammatory properties, where its poor properties were improved by being nanoformulated. The hydrogel was injected into rats that were subjected to surgical ligation of coronary artery to induce infarction. The hydrogel treatment reduced fibrosis, downregulated apoptosis rate and decreased ventricular remodeling [102].

## Conclusion and future perspectives

Nowadays, it is well accepted that the therapy of MI should target cardiomyocyte loss in order to improve patients' prognoses and reduce MI-induced heart failure. Current therapeutic approaches which include surgical interventions or pharmacological agents are not considered as curative treatment. Cardiomyocytes cannot regenerate after necrotic or apoptotic events, and a fibrous tissue eventually forms, reducing myocardial contractility. This has sparked significant interest in cardiac regeneration therapy.

Cell-based regenerative therapy has been widely reported for more than fifty years. However, introducing a commercial cell therapy product has faced major obstacles as cost, regulatory issues and strategic buisness considerations. For cell therapy to be clinically translated, enormous expenses are required in order to combine efficient bioprocessing techniques and scale up approaches which can not be implemented at a cost that the society can afford. Thus, healthcare officials find it a significant burden, particularly in developing countries. Moreover, cell therapy requires evaluation of several factors as cell source, isolation and differentiation methods in addition to purity and potency analysis. It also faces major safety concerns including undesirable in-vivo cell differentiation, migration and tumourigenicity. Recent theories have attributed positive results of cell based therapies to paracrine mechanism. Notably, some studies reported improvement in cardiac repair after administration of cell therapy despite low survival and engrafment rates. Thus, a new mechanism was proposed postulating that the cells secrete cytoprotective factors that contribute to myocardial healing after an ischemic insult. Subsequently, more research interest has been shifted towards cell-free approaches for cardiac regeneration.

Exosomes have attracted attention and augmented the concept of cell-free therapies owing to their biocompatability and target specificity. Exosomes can contribute to the healing of ischemic insult post infarction through modulation of inflammation, fibrosis and angiogenesis. Obtaining patient`s own exosomes renders it more biocompatible and less immunogenic. However, exosomes are biological products that face some challenges as isolation methods. They are mainly isolated according to vesicle size. It was reported that exosomes may contain different products exerting different therapeutic outcomes according to the microenvironment, thus more accurate isolation techniques are required. In addition, they suffer from instability and low retention rates after in vivo administration. Moreover, they need standard operating procedures (SOP) for large-scale isolation and purification. The emergence of exosomes as a successful bioinspired cell-free approach in cardiac regeneration has given an opportunity to introduce more nanoparticles-based strategies capable of targeting infarcted myocardium. Interestingly, these nanoformulations can be prepared and characterized overcoming the challenges associated with isolating and evaluating a biological product. Moreover, these nanoparticles can be designed to target myocardium using different passive or active targeting approaches. These nanoformulations can be loaded with affordable drugs, growth factors or natural products with reported cardioptotective properties. Natural products offer a reservoir of bioactive compounds that can play an integral role in cardiac repair. By optimizing the formulation and surface functionalization of these nanoparticles, we can achieve targeted delivery to the injured heart tissue, enhancing therapeutic efficacy and minimizing side effects. The translation of these nanotechnological advancements into clinical applications holds immense potential. By addressing the limitations of current therapies, such as limited bioavailability and off-target effects, these nanocarriers can offer a more effective and safer approach to cardiac repair. Additionally, the integration of nanotechnology with tissue engineering techniques using injectable biomaterials can further accelerate the development of regenerative therapies for heart disease.

## Data Availability

Not applicable.
